# Impact of Initial Emotional States and Self-Efficacy Changes on Nursing Students’ Practical Skills Performance in Simulation-Based Education

**DOI:** 10.3390/nursrep11020026

**Published:** 2021-04-21

**Authors:** Ricardo Gregorio Lugo, Inger Hjelmeland, Mette Tindvik Hansen, Erna Haug, Stefan Sütterlin, Heidi Kristine Grønlien

**Affiliations:** 1 Faculty of Health and Welfare Sciences, Østfold University College, 1757 Halden, Norway; ricardo.g.lugo@ntnu.no (R.G.L.); inger.hjelmeland@hiof.no (I.H.); mette.t.hansen@hiof.no (M.T.H.); erna.haug@hiof.no (E.H.); stefan.sutterlin@hiof.no (S.S.); 2 Division of Clinical Neuroscience, Oslo University Hospital, 0424 Oslo, Norway

**Keywords:** simulation training, self-efficacy, affective states, expert ratings, nursing skills, nursing education

## Abstract

Training through simulation has shown to increase relevant and specific skills sets across a wide range of areas in nursing and related professions. Increasing skills has a reciprocal relation to the development of self-efficacy. A study was conducted to assess changes in the development of self-efficacy in simulation training for 2nd year nursing students. Initial emotional states, pre and post self-efficacy, and expert ratings of simulation performance were assessed. Results show that students who displayed an increase in self-efficacy as a result of simulation training were also judged to perform better by expert ratings. The effect of simulation on self-efficacy could be influenced by initial states of physiological activation and over control. Results also showed that initial emotional states did not moderate self-efficacy development on outcome measures. These findings improve our understanding on the relationship between students’ self-efficacy and performance of practical skills and inform pedagogical designs and targeted interventions in relation to feedback and supervision in nursing education.

## 1. Introduction

Increased demands for new and more diverse skill sets are changing the professional roles and responsibilities of nursing program graduates. To increase the applicability of theoretical knowledge and the professional preparedness of nursing students, simulation-based education has become an important part of the nursing educational program. Simulation training is described as “activities that mimic reality and variously involve roleplaying interactive videos, or mannequins that help students learn and allow them to demonstrate decision making, critical thinking and other skills” ([1], p. 97). Simulation-based nursing education is a pedagogical approach, helping to expose students to various real-life scenarios and practice their clinical skills [2], and increasing emphasis is placed on the use of simulation and targeted practice in nursing education [3,4]. Simulations provide experiential contexts and outcomes similar to real clinical situations while in a safe and well-controlled environment. These include experiences such as self-observations as well as psychological and physiological reactions to the situational context from both the patient and the nursing student, which can in turn influence the simulated situation and the result in task performance [5,6].

### 1.1. Simulation Training

For students to reach their learning outcomes, several factors in the simulation training need to be present. Ericsson [7] identified that time for decision making and reflection followed by expert follow-up and debriefing alongside opportunities for several paths of action are needed to help consolidate learning. The term deliberate practice entails expert supervised practice (simulation) of specific situations relevant to the domain in order to increase practical skills [7]. In nursing simulation, this method allows novice-nursing students to acquire expertise through both trial and error of procedures accompanied by expert feedback, increasing self-efficacy through direct mastery experience accompanied by feedback (see the section on psychological benefits of simulation below). Research in nursing education has shown that simulation methods accelerate novices’ decision making to achieve higher expert levels [6]. Simulations target Bandura’s [8,9] four process for increasing self-efficacy (see below). Franklin and colleagues’s [10] meta-analysis showed that simulation training improved self-efficacy in nursing students, even though several limitations in most studies were found. However, Franklin and colleagues highlights missing aspects of simulation training’s effects on self-efficacy and that other factors such as self-efficacy must include reliability measurements and reporting of effect sizes.

### 1.2. Simulation and Learning Outcomes

A conceptual framework developed by Jeffries [1] for simulation models in education has become the foundation for how simulations are developed and used. This includes the educational setting and teaching practices as well as how the simulation’s design characteristics (e.g., complexity, cues, debriefing) influence the simulation outcomes such as critical thinking, self-confidence, and skill acquisition. The bidirectional relationship between self-confidence and simulation outcomes (i.e., performance) is currently not well understood. Studies of this relationship might entail relevant consequences for the pedagogical approaches regarding feedback and targeted early supervision intervention. This study will attempt to provide support to the bidirectional relationship by also looking at situational factors (i.e., emotional states) and how they contribute to outcomes.

Simulations facilitate accelerated learning consistent with the deliberate practice paradigm [11]. The ability to control the simulation training allows clinically relevant situations to be adjusted to match suitable levels for participants and thus allow for the development of a more reliable self-assessment. The active learning through simulation under controlled conditions allows for the conscious transfer of theoretical and practical knowledge. For example, Shin and colleagues [12], while examining simulation training over different domains and different levels of nurse expertise reported medium to large pre-post effect sizes (*d* = 0.71) on learning, with the biggest gains for graduate nurses (*d* = 1.16) and nurse practitioners (*d* = 1.06), while nurse foundation levels (undergraduate) had slightly lower, but still medium to large effect sizes (1st year: *d* = 0.49; 3rd year: *d* = 0.86). Other studies have found more inconsistent findings on simulation outcomes. Hegland and colleagues [13] found that computer simulations done in random controlled trials had small effects (SMD-1.09) when compared to other learning strategies.

There are some disadvantages with training through simulation; e.g., factors that would in a real-life situation lead to an increase in anxiety and stress levels can be experienced as awkward, and the simulation scenarios may be perceived as unrealistic or are unlikely to be expected when communicating with patient-simulation mannequins [14]. Students low in confidence have been reported to experience higher stress levels and displayed weak learning outcomes [14]. Therefore, the development of a realistic confidence into one’s own skill (self-efficacy) can be an important condition for good learning outcomes in simulation training.

### 1.3. Psychological Benefits of Simulation Training

Bandura [9] defined perceived self-efficacy as the “beliefs in one’s capabilities to organize and execute the courses of action required to produce given attainments and separated self-efficacy into a specific and a global component”. General self-efficacy relates to the overall belief that one is in control over one’s own life, actions, and decisions that shape one’s life, while specific self-efficacy is the belief into one’s performance in a certain task or described situation. Self-efficacy is also contingent on outcome expectancies, since one must consider the desired outcome and judge if one possesses the skills necessary to reach those outcomes [9]. Self-efficacy can be strong and weak all within one person. A person may be confident in one’s skills in one area of functioning, but that does not automatically generalize to other areas.

Bandura [8,9] identified several influencing factors for perceived self-efficacy. The first source and most prominent if affecting self-efficacy is that of mastery experience. Overcoming any demanding situation in a beneficial way increases the perception of self-efficacy, thus strengthening confidence and self-evaluations, while the opposite happens when failing. Vicarious experience is based on observing a role model perform the same task and then performing the task oneself. The more similar a role model is to oneself, the more sense of self-efficacy one builds, while very dissimilar role models do not induce this sense in observers. Another way of increasing self-efficacy is through social persuasion either by a significant other or by oneself (self-talk). Social persuasion involves instilling beliefs in the person that one can succeed; the point of succeeding in such was that self-affirmation is strengthened and self-efficacy is bolstered. Social persuasion needs to be focused on success in terms of self-improvement rather than by triumph over others. Strengthening self-efficacy can also happen by being in tune with one’s own physiological and emotional states. Interpreting stress reactions and tension has effects on perceiving one’s state and adaptation ability [8,9]. Kavanagh and Bower [15] showed that positive moods improved self-efficacy, while despondent moods decreased feelings of self-efficacy [16]. Looking at emotional aspects of self-efficacy (affective states) has been little researched in simulation training, and this study will address this aspect of self-efficacy. Research has shown that affective states can influence and are influenced by self-efficacy [17,18], and this could lead to targeted interventions to help learning.

Self-efficacy has a reciprocal causal relationship with skill development [9]. When one encounters a problem, initial emotional states and self-efficacy influence how one copes with the situation. Then, a person evaluates how the coping strategy used influences the situation and adjusts strategies accordingly. When one’s skills are not perceived to have an effect on the situation at hand, one can still increase self-efficacy if the cognitive interpretation is focused on learning and not on the outcome itself. On the other hand, by mastering the situation, one will increase the perceptions of self-efficacy. After the situation has ended and the outcomes are experienced, and depending on the interpretations of these outcomes, this then can further influence self-efficacy perceptions.

### 1.4. Findings on Simulation and Self-Efficacy

It has been pointed out that by incorporating relevant educational frameworks into the planned simulation experience gives students the opportunity to engage in meaningful ways of learning about their own and others’ practices and allows novice nursing students to recognize their true self-efficacy [19]. Simulation increases self-efficacy through role-playing in case studies [20]. Simulations support the development of coping strategies for the participants, including handling of personal physiological and emotional changes, the development of cognitive strategies to handle the situation, increased confidence, and reduced stress levels [14]. Pike and O’Donnell [21] reported increased self-efficacy after simulation. A qualitative analysis revealed that communication skills development and more realistic simulations could further improve the confidence gained from the simulations. Simulation has shown to increase self-efficacy in simulations on resuscitation [22], but the computer-based simulation was rated by the students as more satisfying than the mannequin simulation. Tuttle [23] showed that both control groups (school curriculum) and experimental groups (simulations) experienced gains in self-efficacy. A systematic review shows that simulations are effective in establishing learning environments that help learning outcomes and improve confidence [24]. Simulations also allow for the testing of skill that is ecologically relevant for the domain. Simulations achieve ecological validity through modeling real-life scenarios that lead to more skill development, reflection, and critical thinking [25]. More recent studies have used post-graduate interdisciplinary professionals from clinical settings and found that simulation increases confidence alongside communication, leadership, and teamwork skills [26].

However, not all high-fidelity patient simulations have produced positive effects on self-efficacy. Feingold and colleagues [27] reported that less than half (46%) of the students reported an increase in confidence after simulation training, Scherer and colleagues [28] did not find any improvements on self-efficacy between simulation and traditional teaching methods in graduate students, while Karabacak and colleagues [19] reported a decrease in self-efficacy after simulation training. The findings of Scherer and colleagues [28] suggested that students initially low in confidence did not improve. Further research reported unsatisfied participants who perceived the simulation as “not real enough” [22], while Norman [24] highlights that there is still a lack of research providing evidence that simulation can be generalized to clinical situations.

### 1.5. Aim of the Study

The present pilot study aimed to set up a design to investigate the impact on self-efficacy among nursing students in high-fidelity simulation training and further to analyze self-efficacy changes in the context of actual task performance operationalized as intersubjective expert ratings. The aim is also to look at moderating factors’ (emotional states) influence on self-efficacy and performance.

We hypothesize that students’ development in self-efficacy is positively associated with task performance as assessed via expert ratings. We further hypothesize that initial emotional states influence this relationship, as stipulated by Bandura [29].

## 2. Material and Methods

### 2.1. Participants

Participants for this correlational design were recruited from the 2nd year nursing study (Bachelor; *n* = 40) during their spring semester (age range= 21–47; 89% female). The students were acquainted with the simulation structure. All the students had previously, throughout their studies, participated in three similar simulation sequences with pedagogical design based on the International Nursing Association for Clinical Simulation and Learning’s best practice standards [30]. The students were at present in practical studies, and the theoretical underpinnings for the simulation have previously been taught.

### 2.2. Measurements

To measure initial emotional states, a self-assessment manikin (SAM) was used [31] where mood, physiological activation, and control were measured on a 9-point Likert-scale (1 to 9). Previous research has shown the SAM to be valid for both children and adults [32].

To measure situational self-efficacy before and after the simulation, one item was constructed with a modification. Before simulation, participants were asked, “How well do you think you will be able to do on the task?” After simulation, participants were then asked, “How well do you think you did on the task?” following the recommendations by Hoeppner and colleagues [33]. Participants marked a visual analogue scale both before and after the simulation training. The responses were measured from 0 to 100 percent. Hoeppner and colleagues [33] showed that a one item self-efficacy question has better predictive validity than a traditional 10-item scale. One-item scales have shown good reliability and validity when compared to original longer scales [34,35,36], are more adapted to specific situational assessment [34,37], and can be used for rapid assessment [38,39]. Reliability analysis for the two items for this study showed good levels (Cronbach’s α = 0.802).

Expert evaluations consisted of two items based on learning outcomes for each simulation case, as shown in Table 1. The expert rated the students’ performance on a scale from 0 to 100%. The scores were averaged. The experts were four nurses, all with clinical and educational experience of minimum 25 years. Expert ratings in nurse research have been validated as good measures [40]. There was one expert present in each scenario as the actor of the standardized patient in the simulation. Inter-rater reliability was good (Cronbach’s α = 0.869).

### 2.3. Procedure

The scenarios in this study were developed based on descriptions of real situations from the field of practice and in accordance with specification requirements for best practice in simulation [30]. The scenarios were in line with the students’ learning outcomes and validated through testing in previous student cohorts evaluated against the goal of learning outcomes for the simulation; see Table 1 for scenario details. The standardized patients and facilitators were instructed and trained prior to implementation.

Information about the simulation and the pilot study was provided to the students three days prior to the simulation. The information was given through their online learning platform. The information consisted of a specific time schedule of the day and a student version of the scenarios. This student version contained the expected learning outcome, curriculum, and the cases the students were to meet in the different scenarios.

During the initial briefing, the students were divided into groups of five to nine students. The simulation consisted of four different live scenarios where the experts were used as standardized patients. Each group completed all the scenarios once. For each scenario, one student was appointed the role of a nurse and one student was appointed the role as a nurse assistant. Of the remaining students in the scenario, one was given the role of time-taker, and the rest were given the role as an observer. The student’s roles in each scenario changed to include participation as the nurse or the nurse’s assistant so that each student participated as the nurse and nurse assistant one time. Otherwise, students served as time-takers or observed the scenario and provided feedback in the debriefing sessions.

The observers and the time-taker first entered the room. They found their designated places around a table where they had a good view of the scene of simulation. Before each scenario, the “nurse” and the “nurse assistant” filled out their emotional states on the SAM and rated their situational self-efficacy. Then, they entered the room and the simulation started. Each simulation sequence lasted approximately 20 min (10 min used on roleplaying and 10 min for debriefing). Table 1 describes the situation, required action, learning outcomes, and learning expert evaluation items of the scenarios. In each scenario, after roleplaying, but before debriefing took place, the students were asked to re-evaluate their self-efficacy. Their scores were based on their own impressions of their performance.

### 2.4. Analysis

Statistical analysis was done with IBM SPSS software Version 24 (IBM Corp., Armonk, NY, USA). All variables were centered and standardized for analysis. Alpha (α) levels for hypothesis testing were set at the 0.05 level (two-tailed). The dependent variable Task performance was operationalized as the two averaged expert ratings. A change variable for self-efficacy (SE) was computed (SE change = SE post-SE pre). To assess the simulation-induced self-efficacy change’s influence on the dependent variable performance, a hierarchical regression was conducted in which initial emotional states (visual analogue scales on SAM) were entered in the first step, and the change in self-efficacy was entered in the second step. To test for moderation effects of emotional states and self-efficacy on task performance, moderation analysis (PROCESS) was adopted from Hayes [41] and follows the guidelines established by Baron and Kenny [42], and a hierarchical multiple regression analysis was conducted. In the first step, both predictor variables (emotional states and self-efficacy change) were included. In step 2, an interaction variable for the independent variables was created and added to the regression. Since the first step of regression yielded a significant result and the resulting change in explained variance due to the interaction is significant, a moderation effect of the predictor variables was tested for. A post hoc power analysis (G*power) [43] revealed that a sample size of 55 would be needed to achieve significant power.

### 2.5. Ethics

The study conformed to institutional guidelines and was eligible for automatic approval by the Norwegian Social Science Data Services’ (NSD) ethical guidelines for experimental studies. After the initial NSD online application was filled in, no further formal applications were required after Norwegian law, since only pseudonymized and non-health related data were collected and processed. Participants gave their informed consent verbally prior to the study and were debriefed about the study’s purpose after completing the data collection. Participants were informed that they could withdraw from participation at any time and without any consequences throughout and after the session.

## 3. Results

Descriptives and correlations for all items are displayed in Table 2 and Table 3. The expert scale reliability for the two expert ratings across all scenarios was strong with ⍺ = 0.869. Both ratings were averaged to create a score for task performance. In this study, nine respondents were excluded from the analysis, since the role of nurse assistant was too passively played to be able to set an expert score. In addition, two students never participated as a nurse/nurse assistant. If the respondent participated more than once as a nurse/nurse assistant, only the first time was included in the analysis.

To test the hypothesis on changes in self-efficacy, two analyses were performed. First, a paired sample t-test was performed to see if the simulation increased self-efficacy at the group level. To test if the simulation increased individual levels of self-efficacy, a change in self-efficacy (SE change) was computed (SE post–SE pre). A hierarchical regression analysis where initial emotional states (SAM) were added as independent variables in the first step and self-efficacy change (SE change) was added in the second step, and averaged expert ratings as the dependent variable was performed.

**Figure 1:**
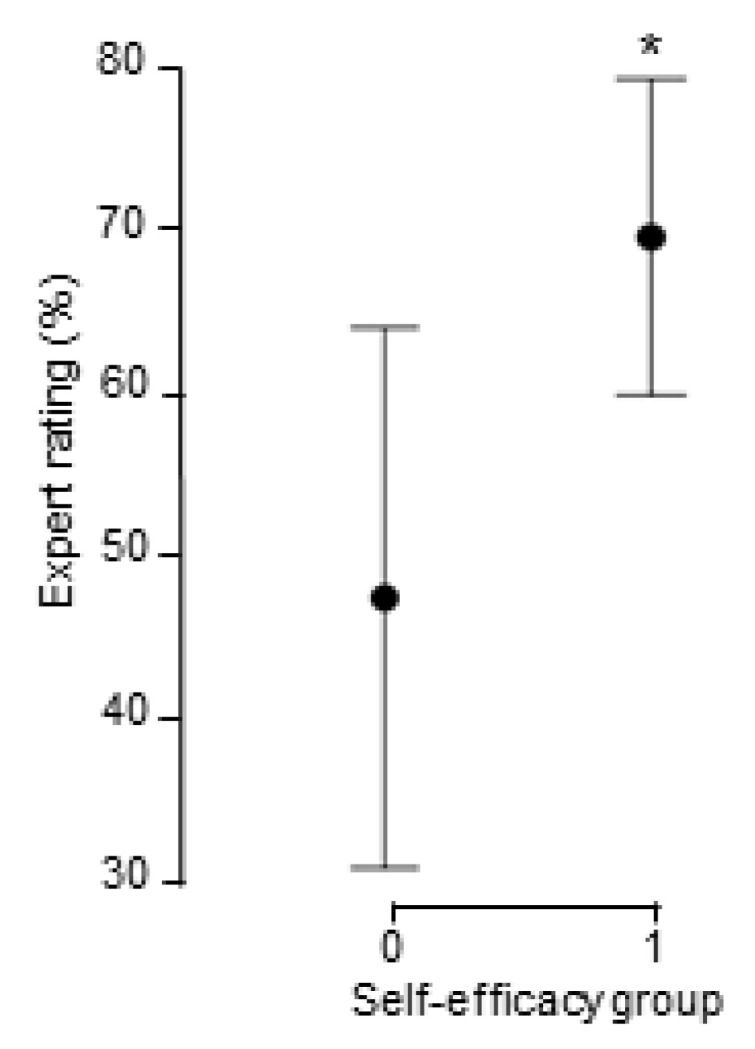
Group differences in self-efficacy change and expert ratings. The data are presented as means with 95% CI error bars. The self-efficacy group 0 represents the respondents with a negative change in self-efficacy, while group 1 represents the respondents with a positive change. * indicates a significant higher mean value at 0.05 level.

On group level, there was no significant increase in self-efficacy (*t* = 1.12, *df* = 27, *p* = 0.274, Cohen’s *d* = 0.17) after simulation training. For individual scores, we conducted a regression analysis where both the affective state measurement and self-efficacy change variable were used as predictors. The results show that initial emotional states were not associated with expert ratings (*F*(3,23) = 1.40, *p* = 0.268, *R^2^_adj_* = 0.040), but that positive changes in self-efficacy during simulation predicted better performance scores (*F*(4,22) = 3.01, *p* = 0.040, *R^2^_adj_* = 0.236). Change in self-efficacy was the only significant predictor (*t* = 2.60, β = 0.547, *p* = 0.016) of expert performance. To further explore the hypothesis of how self-efficacy changes are affected by simulation performance, two groups were created: one group who reported positive changes in self-efficacy and the other group who reported negative changes in self-efficacy. Then, they were compared to each other in relation to expert evaluation. Results showed that students who reported a positive change (*n* = 12, *M* = 69.58 *SD* = 12.29) in self-efficacy were better rated than students who reported a negative change (*n* = 15, *M* = 47.67, *SD* = 29.93, *t* = 2.46, *df* = 21.69, *p* = 0.022, Cohen’s *d* = 0.89, Figure 1).

To test the moderating effects of affective states on self-efficacy, initial emotional states measured by the SAM did have an effect on changes in self-efficacy, where an initial physiological activation correlated positively with self-efficacy changes (*r* = 0.332, *p* = 0.042), while control was strongly negatively correlated (*r* = −0.627, *p* < 0.001). To see the effects that initial emotional states had on changes in SE, a regression analysis was calculated and found that initial states were significantly associated with SE changes (*F*(3,24) = 5.23, *p* = 0.006), but of these emotional state variables (mood, physiological activation, control), only initial control (β = −0.658, *p* = 0.003) was significant and accounted for 27% of the variance in SE change (*r* = −0.520). Then, a moderation analysis was conducted to test the interaction of self-efficacy change and initial emotional states on the dependent variable (Task performance). No significant moderation effects were found (Δ*R^2^* = 0.064, Δ*F* = 2.30, *p* = 0.143), indicating that initial emotional states did not moderate the relationship between self-efficacy and task performance.

## 4. Discussion

This pilot study set out a design to see how self-efficacy changes during simulation would be reflected in expert ratings and how initial emotional states can moderate this process. Results show positive support for the hypotheses. At the group level, self-efficacy did not significantly increase as a result of the simulation. However, positive changes in self-efficacy were associated with better performance in simulation training, as assessed via expert ratings, but initial confidence levels expressed as perceived control had a debilitating effect on self-efficacy.

Initial emotional states did not moderate self-efficacy development, contrary to Bandura’s explanation [9] that situational self-efficacy may be a better predictor for performance and that initial affective states must be taken into consideration. While heightened physiological arousal predicted better performance as found in previous studies regarding arousal and performance [44,45], emotional control showed negative associations with self-efficacy changes in this study. While studies on physiological arousal and heightened performance are abundant, situational over-control, as found in this study, might be related to maladaptive perfectionism [46]. Over-control has also been found to be associated with perfectionism, which predicts lower self-efficacy [47]. For better understanding of these associations, future research on the role of self-efficacy in nursing simulation should include perfectionism.

These findings add to the accumulating literature that simulation training may increase self-efficacy but only at the individual level, as there were no findings at the group level [20,21,22,24,48,49].

The results show an increase in certain individuals and that overconfidence can moderate these changes, but it is difficult to predict which individual would benefit from this specific simulation. Factors such as age, gender effects, or other cognitive factors were not measured in this study. Feingold and colleagues [27] and Scherer and colleagues [28] found no effects for students low in confidence, but these studies did measure group differences. Simulations require a good user fit for learning, and not all simulations may elicit interaction from the participants, just as not all subjects fit with all students. Therefore, it would be better to measure changes at the individual level rather than the group level. Unlike previous research, this study assessed task performance via expert ratings. The experts have several years of clinical experience, and they were involved in the conceptualization of the simulation. Their expertise and involvement in the simulation was meant to diminish the “unrealness” that was previously reported [22,25]. In addition, by using expert ratings on performance, the reported changes in self-efficacy are not just subjective pre/post reports but corroborated by intersubjective performance measurements. While the expert evaluations were not directly related to self-efficacy, they implicitly assessed coping strategies for the situation. These measurements include a correct decision choice and confidence in the strategy chosen by implementing appropriate actions. If participants did not believe they could solve the problem, this can have impacted their decisions and implementation ratings.

These results cannot specifically identify which of the possible factors outlined by Bandura [9] caused the observed effects. However, some valuable conclusions can be made to stimulate future research. This simulation involved neither role modeling nor verbal persuasion. Instead, it consisted of an initial briefing followed by the task and subsequent debriefing. Since the only other processes remaining are direct experience and concomitant physiological changes, it can be inferred that the simulation increased self-efficacy through these processes. As Bandura [9] outlined, direct experiences are the strongest source of increasing self-efficacy, and the experience of physiological reactions are the weakest. However, developing self-efficacy works on all those levels (role modeling, verbal persuasion, physiological feedback). This may also explain why group effects were not present. Students were either part of the simulation or observing, and while the simulation increased self-efficacy only in individuals, but not at the group level, positive mastery experiences were associated with better expert ratings.

There are several limiting factors to this pilot study. The power for our sample size (*n* = 40; β = 65%) is too small for achieving statistical power at β = 80%, and this can lead to type I and type II errors. The findings from this simulation cannot be generalized to clinical settings, as described earlier [22,27,28]. Simulations involved more than one active participant, but macro cognitive factors were not included. Klein et al. [50] have shown that team factors such as communication and coordination help situations and outcomes and thus may also increase self-efficacy. The study does not have matched controls and is associative in nature; therefore, causality cannot be assumed even though changes in self-efficacy were measured before debriefings on expert evaluations. The simulation did not produce group effects. This makes it difficult to understand if the simulation was too difficult, since only inter-individual levels changed. This may be because student had been previously exposed to simulations in their educational track. In this natural education setting, the individual participants were exposed to different scenarios with 40 students distributed over four different scenarios. Students who participated as a nurse or nurse assistant more than once only had their initial simulation counted in the analysis. Some individuals were exposed to difficult tasks, thus adding to task-dependent inter-individual differences, which might partially explain both self-efficacy gains and non-gains. One final limitation is that the scenarios used in this study was done with live actors, while previous research reviewed has used virtual patients or mannequins, which may lead to misinterpretations when comparing to previous results.

The results from the study have implications for future research. Studies should include repeated measurements of situational self-efficacy across scenarios of various difficulty levels. While these findings cannot be generalized to clinical settings, the inclusion of expert judgments rather than participant self-reports would give better indications of performance since they are more objective in nature, are relevant to learning outcomes, and can be adapted by reflecting with clinical settings and the factors that are relevant for healthcare outcomes. The measurements proposed here (situational self-efficacy, emotional states, expert ratings) should complement other macro cognitive factors (i.e., team workload demands, communication, cooperation) as described by Watters and colleagues [26], alongside other human factor approaches and decision-making styles that may be relevant in comparable high-stake situations (perseverative cognition, metacognition, perfectionism).

## 5. Conclusions

The purpose of the present study was to investigate the impact of initial emotional states and changes in self-efficacy during simulation training among nursing students. Students who showed an increase in self-efficacy as a result of simulation training performed practical skills better. These findings improve our understanding of the relationship between students’ self-efficacy and the performance of practical skills and inform pedagogical designs and targeted interventions in relation to feedback and supervision in nursing education. While initial emotional states in this study did not moderate the effect of self-efficacy on simulation results, future research should include the initial emotional states to elucidate the influence simulation-based education has on the development of self-efficacy. The study contributes to the simulation-based education by identifying reliable and validated tools and triangulate the findings using expert assessments and not just subjective reports. These results can help future simulation designs for more accurate measurements and further investigate other cognitive determinants of performance.

## Figures and Tables

**Table 1:** Scenario descriptions.

Scenario	Required Action *	Learning Outcomes	Expert Evaluation Items
Preoperative	Information consultation before operation	Skills: Pre-operation skin care. Post-operation procedure, information about mobilization, elimination, pain, and nausea care	How successful was the student to provide information on skin preparation before the impending surgery? How successful was the student in providing information on the post-operative phase?
Postoperative	Observation and nursing measures	Knowledge: Pain evaluation NRS scaleSkills: Intravenous infusion administration for pain and nausea	How successful was the student in assessing the treatment of pain and nausea? How successful was the student in managing pain and nausea intravenous treatment?
Mobilization	Help the patient out of bed, walk a few steps and back into bed again	Knowledge: Pain prevention increasing patient activity Skills: Patient assistance through mobilization	How successful was the student at preventing pain when the patient was getting out of bed? How well did the student collaborate so that the patient finds the best way to get to the bedside?
Sepsis	Observation and nursing measures	Knowledge: Observation for suspicion of infection and sepsis development Skills: Blood culture orders and physician consultation	How well did the student control the identity and the blood transfusion form? How successfully did the student perform safe blood management using the right equipment?

* Evidence-based healthcare procedures retrieved from VAR Healthcare https://www.varhealthcare.com/ (accessed on 31 May 2020).

**Table 2:** Descriptive statistics of SAM, SE, and Expert ratings.

	Minimum	Maximum	Mean (M)	Standard Deviation (SD)
SAM Mood	2	9	5.55	1.68
SAM Activation	2	9	6.66	1.65
SAM Control	1	8	4.69	1.83
SE pre	20	90	58.07	16.61
SE post	10	90	61.64	21.18
SE change	−50	50	6.20	27.98
Expert score 1	0	100	55.56	27.64
Expert score 2	0	90	59.26	28.81
Expert average	5	90	57.41	26.54

Abbreviates: SAM, self-assessment manikin; SE, self-efficacy. *n* = 29.

**Table 3:** Bivariate correlations between items of SAM, SE, and Expert Ratings.

	2	3	4	5	6	7
1. SAM Mood	−0.353	0.451 *	0.417 *	0.196	0.098	−0.223
2. SAM Activation		−0.602 **	−0.337	−0.066	0.228	0.332 *
3. SAM Control			0.396 *	−0.079	−0.288	−0.627 **
4. SE pre				0.686 **	−0.241	−0.073
5. SE post					0.261	0.639 **
6. Expert Average						0.541 **
7. SE change						

Abbreviates: SAM, self-assessment manikin; SE, self-efficacy. *n* = 29; * *p* < 0.05 (2-tailed), ** *p* < 0.01 (2-tailed).

## Data Availability

The data presented in this study are openly available in Mendeley Data at doi:10.17632/rkntd694sf.1.
